# Exercise and Motor Training in People with Parkinson's Disease: A Systematic Review of Participant Characteristics, Intervention Delivery, Retention Rates, Adherence, and Adverse Events in Clinical Trials

**DOI:** 10.1155/2012/854328

**Published:** 2011-11-16

**Authors:** Natalie E. Allen, Catherine Sherrington, Gayanthi D. Suriyarachchi, Serene S. Paul, Jooeun Song, Colleen G. Canning

**Affiliations:** ^1^Clinical and Rehabilitation Research Group, Faculty of Health Sciences, The University of Sydney, P.O. Box 170, Lidcombe, Sydney, NSW 1825, Australia; ^2^Musculoskeletal Division, The George Institute for Global Health, The University of Sydney, Sydney, NSW 2050, Australia

## Abstract

There is research evidence that exercise and motor training are beneficial for people with Parkinson's disease (PD), and clinicians seek to implement optimal programs. This paper summarizes important factors about the nature and reporting of randomized controlled trials of exercise and/or motor training for people with PD which are likely to influence the translation of research into clinical practice. Searches identified 53 relevant trials with 90 interventions conducted for an average duration of 8.3 (SD 4.2) weeks. Most interventions were fully supervised (74%) and conducted at a facility (79%). Retention rates were high with 69% of interventions retaining ≥85% of their participants; however adherence was infrequently reported, and 72% of trials did not report adverse events. Overall, the labor-intensive nature of most interventions tested in these trials and the sparse reporting of adherence and adverse events are likely to pose difficulties for therapists attempting to balance benefits and costs when selecting protocols that translate to sustainable clinical practice for people with PD.

## 1. Introduction

In recent years there have been an increasing number of randomized controlled trials assessing the effects of exercise and/or motor training in people with Parkinson's disease (PD). Overall, these trials support exercise and motor training as beneficial in improving walking, balance, muscle strength, and the performance of functional tasks in people with mild-to-moderate PD [1–11]. In order for findings from this research to be of general benefit to people with PD, therapists need to be able to translate the protocols used in the research into clinical practice [12].

Evidence-based practice aims to incorporate and apply high-quality clinical research findings in clinical policy and practice [13, 14]. However, this can be a challenging task as health practitioners may find it difficult to assess, interpret, and implement research evidence [13]. While evidence about beneficial outcomes is paramount in therapists' decisions to implement a particular intervention, there are other factors that affect how the overall impact of the intervention is interpreted and its potential for widespread clinical application [13–17]. For example, therapists need to consider how the characteristics of participants included in a trial may affect their decision regarding the applicability of the trial intervention with their patients [14]. It is likely that the way in which the intervention was applied in terms of its duration, level of supervision, delivery (i.e., individual versus group), and location (e.g., facilities and equipment required) will influence therapists' decisions to implement that intervention. A research protocol that has been shown to be effective may not be implemented by therapists if they cannot provide adequate supervision over the required time frame or they do not have access to necessary facilities or equipment. Finally, information regarding retention, adherence, and adverse events is required so that therapists and patients can weigh up the effectiveness of the intervention against its acceptability and any risks associated with implementation [14]. 

Therefore, in order to examine the information available to guide the translation of research into clinical practice, we searched randomized controlled trials of exercise and/or motor training for people with PD to determine the

1. disease severity and cognitive status of the included participants,
2. duration, supervision, delivery, and location of the interventions,
3. rates of retention, adherence, and adverse events.

## 2. Methods

### 2.1. Data Sources and Searches

Randomized controlled trials of exercise and/or motor training for people with PD were identified via database searches of MEDLINE, EMBASE, AMED, PsycINFO, the Cochrane Central Register of Controlled Trials, and CINAHL. The initial search was conducted in 2009, with a subsequent search conducted over 5 days from the 7th of April, 2011. The electronic search strategy used has been previously reported [2]. The Physiotherapy Evidence Database (PEDro; http://www.pedro.org.au/) was also searched, and the reference lists of previously published systematic reviews [4, 5, 8, 9, 18–30] were checked for any trials not identified with the database search.

### 2.2. Study Selection

Trials included were published randomized (or quasi randomized, i.e., not truly random but intended to produce similar groups, such as allocation by odd and even birth dates [31]) controlled trials of people with PD where at least one of the interventions was an ongoing program of exercise and/or motor training. All forms of exercise (e.g., aerobic, strength, and treadmill walking) and motor training (e.g., cueing and movement strategy training) were included. Whole-body vibration was not considered to be exercise or motor training. Trials were excluded if the intervention was multidisciplinary or was primarily occupational therapy.

The eligibility of trials was determined in a two-stage process. Firstly, all trial titles and abstracts were screened independently by two investigators (N. E. Allen and G. D. Suriyarachchi). Trials were excluded if it was clear that they did not meet the inclusion criteria. Secondly, the full article was obtained for the remaining trials and each trial was assessed independently by at least two investigators (N. E. Allen, C. G. Canning or J. Song), using a standardized form containing the details of the inclusion criteria. Care was taken to identify trials that had been reported in more than one journal article. Where this occurred, the multiple articles were counted as one trial and all articles were used to collect data for that trial.

### 2.3. Data Extraction

A data collection form was developed, tested on five randomly selected trials and then modified accordingly. All investigators were involved in data extraction, and all data was double-checked by an investigator not involved in its initial extraction (N. E. Allen or J. Song). Discrepancies were resolved by discussion.

Information extracted from each trial included a description of participants (including cognitive status), details of the exercise and motor training program and how it was administered, as well as details regarding retention rates, adherence to the intervention, and monitoring and reporting of adverse events. Retention was defined as the number of participants who completed the trial (i.e., undertook the first or only post-intervention assessment excluding further follow-up assessments) expressed as a percentage of the number of participants who began the trial. Adherence was defined as the number of intervention sessions attended by participants expressed as a percentage of the number of intervention sessions prescribed [15].

## 3. Results

**Figure 1:**
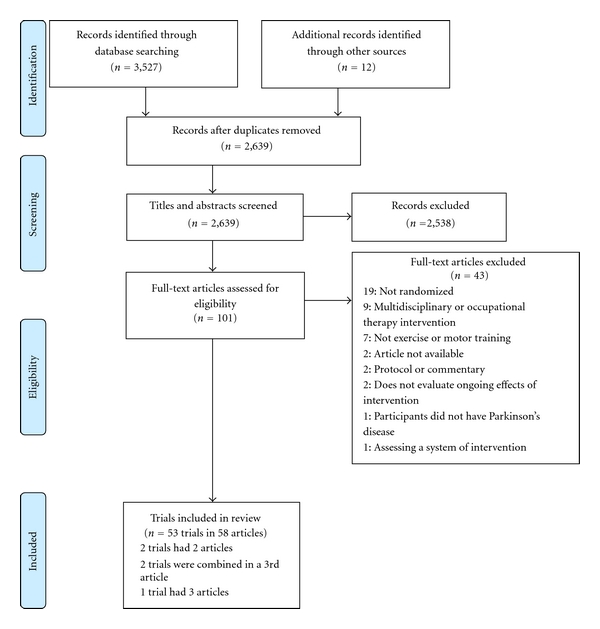
PRISMA flow diagram [32] showing flow of information through the review.

Searching identified 3,539 records, of which 53 trials involving 1,940 participants were found to be eligible for inclusion in the paper (Figure 1) [32]. There were no disagreements between reviewers regarding the inclusion of any articles. The characteristics of the included trials [1, 3, 6, 7, 10, 11, 33–85] are summarised in Table 1.

### 3.1. Participant Characteristics

Forty (75%) of the reviewed trials included participants with mild-to-moderate PD (i.e., equivalent to Hoehn and Yahr stage I to III [86]). Seven trials (13%) included participants with mild-to-moderately severe PD (i.e., Hoehn and Yahr stage I to IV), while four trials (8%) included only participants with mild PD and two trials (4%) included only participants with moderate PD (Table 1). Most trials stipulated the cognitive status of included participants. Twenty-nine trials (55%) used the Mini-Mental State Examination [87] to screen potential participants' cognitive abilities, with the minimum score for inclusion varying between 20 and 28 out of the maximum of 30 [1, 3, 6, 7, 10, 11, 34, 36, 39, 41–43, 45, 47–50, 56, 58, 60, 63–65, 69, 71, 76–78, 80, 85]. One trial (2%) [70] specified that participants required at least moderate scores on the Neurobehavioural Cognitive Status Examination [88]. Thirteen trials (25%) made a statement to the effect that included participants had no dementia and/or reasonable cognition [35, 46, 51, 52, 57, 59, 62, 66, 67, 79, 81, 83, 84]. Ten trials (19%) did not give a clear indication of the participants' cognitive abilities [33, 37, 40, 53, 55, 68, 72, 74, 75, 82].

### 3.2. Exercise and/or Motor Training Program Characteristics

In the 53 trials, there were 90 intervention groups that involved exercise and/or motor training (including two intervention groups for the cross-over trials where one intervention was a control [11, 42, 47]) (Table 1). Average intervention duration was 8.3 weeks (SD = 4.2, range = 2 to 26 weeks), with 37 trials (70%) conducting an intervention of 10 weeks or less. The total number of hours of intervention was not clearly reported in all studies (see Table 1); however, from the available data, an average of approximately 20 hours (SD approximately 11, range = 4 to 65 hours) appears broadly representative of the included trials. Sixty-seven of the 90 intervention groups (74%) involved full supervision of exercise and/or motor training. Participants in 18 (27%) of the fully supervised intervention groups received one-on-one supervision and 20 (30%) received supervision in small groups but the intervention delivery (one-on-one or small group supervision) was unclear in the remaining 29 (43%) intervention groups. Participants in most intervention groups (71; 79%) were required to attend a facility for all or the majority of the intervention sessions.

### 3.3. Retention, Adherence, and Adverse Events

Retention was generally well reported and was high, with 62 (69%) of the 90 intervention groups retaining at least 85% of participants (Table 1). Seventeen (32%) of the 53 included trials reported that at least one participant dropped out for a reason related to the intervention (Table 2). Difficulties with transport and disinterest/poor adherence were the most common intervention-related reasons for dropouts. 

Overall, adherence and adverse events were infrequently reported in the included trials (Table 1). Adherence was reported in some form in 26 (49%) of the included trials. However, 11 (42%) of these trials only reported adherence for those participants who completed the intervention. Most trials (38; 72%) did not report monitoring for adverse events. Across the remaining 15 trials, 11 adverse events occurred (Table 1). Four participants from two separate trials [41, 80] experienced cardiac problems. Two of these participants, one from each group in a trial comparing physical therapy with and without mental practice [80], withdrew from the study. The two participants from the other trial [41] were able to continue safely with treadmill training. Other adverse events reported included a fall [81] and muscle cramps and tiredness [43] in trials involving cued overground walking, knee pain during a dancing program [52], muscle soreness and shoulder pain [56] following resistance training, and a hernia [57] subsequent to muscle strength assessment.

## 4. Discussion

A substantial number of randomized controlled trials of exercise and/or motor training for people with PD have been published. However, the nature and reporting of these trials are likely to provide challenges for therapists aiming to implement the interventions into clinical practice [17]. Most trials involved only cognitively intact participants with mild-to-moderate PD. Trials tended to be of short duration, highly supervised, and conducted at a facility. Furthermore, the reports for many trials were lacking important details, with adherence and adverse events particularly being inadequately reported. 

On the whole, trials included in this paper included only participants with mild-to-moderate PD who were without significant cognitive impairment. Including only these types of participants not only makes it easier to conduct trials of exercise and motor training interventions but also aids interpretation of the results. However, cognitive impairment is now recognised as a common problem in PD, with over 80% of people with PD ultimately developing dementia [89]. Further work is needed to determine the effectiveness of exercise and motor training in people with more severe cognitive impairment and/or more advanced disease.

Most of the reviewed trials were of short duration, highly supervised, and facility based (Table 1). Interventions lasted an average of around two months. Seventy-four percent of the intervention groups were fully supervised, with no reported expectation for participants to undertake unsupervised exercise. Furthermore, 79% of intervention groups were mainly conducted at a facility such as a hospital or university. Such brief, highly supervised interventions conducted in controlled environments are likely to improve the adherence of participants to exercise and motor training programs and to ensure that interventions are being performed optimally. In this regard, these trials are useful and important for determining the short-term efficacy of an intervention. However, given that PD is a long-term, neurodegenerative condition, the capacity of therapists and patients to sustain the intervention over the long term needs to be considered. Furthermore, such brief and highly supervised interventions are costly and less likely to give information about the effectiveness of the intervention when implemented into usual practice [13, 17]. For example, the requirement for participants to travel to a facility was a common reason for withdrawal from the included trials (Table 2). Moreover, the neurodegenerative nature of PD and the limited resources available to healthcare systems mean that such labor-intensive programs are unlikely to be sustained or afforded by most health-care providers. Additionally, as PD is a progressive disease it is important that people with PD are empowered to self-manage their disease to some extent [90, 91]. To this end, trials of more pragmatic and sustainable exercise and motor training interventions, with the potential for direct translation into clinical practice and including cost-effectiveness analysis, are needed.

The likely adherence to an exercise and motor training program is an important factor to consider when prescribing such a program for an individual with PD. Adherence to the intervention was reported in less than half of the included trials, and some reports of adherence are artificially elevated by including only those participants who completed the trial (Table 1). Some trials were able to effectively maximise adherence by providing a flexible timeframe for participants to complete the intervention [46, 51, 52, 74, 76] and so allow participants more options in fitting their exercise and/or motor training program around their daily lives. This pragmatic approach is likely to more closely reflect therapy attendance patterns and is therefore likely to be helpful for therapists considering translating the research into their clinical practice.

Given the importance of adherence to exercise and motor training programs, strategies to promote adherence in people with PD need to be considered. Providing a high level of supervision seems likely to promote adherence in the short term, as it may enhance participants' commitment to the program. However, a Cochrane review comparing home and centre-based exercise programs for older adults found that, in the long term, participants were more likely to adhere to home-based programs [92]. Furthermore, the reviewers noted a trend toward more sustained improvements in the home-based than in the centre-based programs and suggested that this was attributable to the higher adherence in home-based programs. In the present paper, three of the included trials report high levels of adherence with minimally supervised home-based programs [40, 43, 81]. Common to all three of these trials was a requirement for participants to keep a daily record of what exercise/motor training they had performed. It seems likely that this simple strategy assisted in promoting adherence in these trials. Other strategies with the potential to improve adherence in sustainable, minimally supervised trials, such as participant involvement in goal setting [93, 94], flexibility to allow programs to be modified for individuals [1, 91, 93, 94], and intermittent followup [91, 94], warrant exploration.

The issue of adverse events was inadequately addressed in the trials included in this paper, with only 15 trials reporting monitoring for adverse events. In these 15 trials, 11 adverse events were reported, most of which were minor in nature (Table 1). However, when discussing and planning exercise and motor training options with people with PD, therapists need to be informed not only about the effectiveness of a given intervention but also about the nature and likelihood of any potential adverse events [95]. Similarly poor reporting of adverse events was found in a recent Cochrane review of progressive resistance training for older adults [95]. Notably, the Cochrane review found that adverse events were more likely to be detected in trials that used a clear definition of adverse events than in trials which did not use a definition. In the same way, the use of a definition for adverse events is likely to improve the assessment and reporting of adverse events in trials of exercise and motor training for people with PD.

This paper has examined several factors in the nature and reporting of trials of exercise and/or motor training which are likely to influence the way research is applied by therapists in clinical practice. However, this paper did not address whether or not trial protocols were reported in sufficient detail to allow therapists to emulate the research intervention in the clinic. This detailed reporting of trial interventions is critical in enabling research to be clinically applied [96]. The ability of many journals to provide online material which supplements the published article will aid the provision of such details despite the necessary word count limitations placed on authors.

## 5. Conclusions

Clinicians seeking to use research to inform their clinical practice rely heavily on the design and reporting of randomized controlled trials to reach their decisions. However, the nature and reporting of trials of exercise and/or motor training for people with PD are likely to provide challenges for therapists aiming to implement the interventions into clinical practice. The short duration, highly supervised and facility-based nature of many of the interventions, coupled with the tendency to include only cognitively-intact participants with mild-to-moderate disease, mean that findings may not generalise when therapists set out to apply them in the long-term management of people with PD. Infrequent reporting of adherence and adverse events compounds this problem and makes cost-benefit balancing more difficult. It is recommended that these issues be taken into account in the design and reporting of future trials.

## Figures and Tables

**Table 1:** Characteristics of the 53 included trials.

First author, year and intervention type	Initial group sizes	Disease severity	Location and delivery of experimental intervention	Duration of intervention (weeks)	Hours of intervention (approx)	Supervision (%)	Retention (%)	Any dropouts related to intervention?	Adherence (%)	Adverse events occurred
Allen 2010,		Mild to	Facility, group							
exercise OR	24	moderate	+ home individual	26	65	10%	87.5%	Y	70%	N
control	24						100%			

Ashburn 2007,		Moderate	Home,							
exercise and	70		individual	6	33	18%	96%	Y	U (99% of Supervised exercise)	N
strategy OR										
control	72						92%			

Bergen 2002, aerobic exercise OR		Mild	Facility,					NA	NR	NRM
	4		delivery	16	32	100%	100%			
			unclear							
control	4						100%			

Blackinton 2002,	U	Mild to	Facility,				U		NR	NRM
exercise OR	15	moderate	group + home	6	15	66%	53%	Y		
control	total		individual				total			

Bloomer 2008,		Mild	Facility,							
resistance	8		delivery	8	8	100%	75%	N	U	N
training OR			unclear							
control	8						88%			

Braun 2011,		Mild to	Facility,						U	NRM
physiotherapy		moderately	individual or		6 + home					
+ mental	25	severe	group + home,	6	mental	U (100%)^‡^	88%	Y	(87%)^†‡^	
practice OR			individual		practice					
physiotherapy + relaxation	22			6	6 + home relaxation	U (100%)^‡^	82%		(88%)^†‡^	

Bridgewater		Mild to	Facility, group					NA		NRM
1996 (also as [38]),		moderate								
aerobic and	13			12	16 to 22	100%	100%		95%	
trunk muscle training OR										
education	13			12	4		100%		U	

Burini 2006*,		Mild to	Facility, group							NRM
aerobic	13	moderate		7	17	100%	85%	Y	87%	
exercise OR										
Qigong	13			7	17	100%	85%	Y	88%	

Caglar 2005,		Mild to	Facility, group					NA		NRM
exercise OR	15	moderate	+ home,	9	63	4%	100%		100%	
control	15		individual							

Cakit 2007,		Mild to	Facility,						NR	Partial—
treadmill	27	moderate	delivery	8	8	100%	78%	U		2 minor
walking OR			unclear							cardiac
control	27						37%			

Comella	U	Mild to	Facility,				U			NRM
1994*,	18	moderate	delivery				89% total			
physiotherapy	total		unclear	4	12	100%		U	U	
OR										
control										

de Bruin 2010,		Mild to	Home,							
walk with	16	moderate	individual	13	19.5	0%	69%	Y	100%^†^	Y—1
music OR										thigh
control	17						65%			cramping
										1 tiredness

Dereli 2010,		Mild to								NRM
supervised	16	moderate	Facility,	10	22.5	100%	94%	Y	100%^†^	
exercise OR home exercise			individual;							
	16		home, individual	10	22.5	4%	94%		NR	

Dias 2005,		Mild to	Facility,					NA		NRM
physiotherapy	8	moderate	delivery	4 to 10	20	100%	100%		100%	
+ cues OR			unclear							
physiotherapy	8			4 to 10	20	100%	100%		100%	

Ebersbach 2010,		Mild to moderate							NR	NRM
LSVT BIG	20		Facility, individual;	4	16 + home X	U (100%)^‡^	100%	NA		
OR Nordic walking	20		facility, group;	8	16 + home X	U (100%)^‡^	95%	U		
OR home exercise	20		home, individual	4	U	U	95%	N		
			+ all did home, individual							

Ellis 2005*		Mild to	Facility, group							NRM
(also as [44]),		moderate								
physiotherapy	35			6	18	100%	91%	N	93%	
OR										
control	33						94%	N		

Fisher 2008,		Mild	Facility,					NA	NR	N
treadmill	10		delivery	8	18	100%	100%			
walking OR			unclear							
physiotherapy	10			8	18	100%	100%			
OR										
control	10						100%			

Frazzitta 2009,		Moderate	Facility,					NA	NR	NRM
Treadmill	20		individual	4	9.5	100%	100%			
walking with										
cues OR										
physiotherapy	20			4	9.5	100%	100%			
with cues										

Guo 2009,		Mild to	Facility, group					NR	NR	NRM
physiotherapy	23	moderate	education and individual	8	12 of therapy	100%	91%			
and education			therapy							
OR										
control	21						90%			

Hackney 2007,		Mild to	Facility,					NA		NRM
Tango OR	9	Moderate	group	13	20	100%	100%		100%	
exercise	10			13	20	100%	100%		100%	

Hackney 2008		Mild to	Facility,							NRM
(also as [54]),		moderate	group implied							
Tai Chi OR	17			10 to 13	20	100%	76%	Y	100%^†^	
control	16						81%			

Hackney 2009		Mild to	Facility,							Partial—
(also as [54]),		moderate	group							1 knee
Waltz/foxtrot	19			10 to 13	20	100%	89%	Y	100%^†^	pain
OR										
tango OR	19			10 to13	20	100%	74%	Y	100%^†^	
control	20						85%			

Hackney 2010,		Mild to	Facility, group							NRM
partnered	19	moderate		10	20	100%	79%	Y	100%^†^	
tango OR										
nonpartnered	20			10	20	100%	80%	Y	100%^†^	
tango										

Hass 2007,		Mild to	Facility,					NA	NR	Y—1
resistance	10	moderate	delivery	12	12	100%	100%			muscle
training +			unclear							soreness
supplement										and 1
OR										Shoulder
resistance	10			12	12	100%	100%			pain
training +										
placebo										

Hirsch 2003,		Mild to	Facility,							
balance and	9	moderate	delivery	10	22.5	100%	67%	Y	89%^†^	Y—1
resistance			unclear							inguinal
training OR										hernia
balance	9			10	15	100%	100%		92%	
training										

Keus 2007,		Mild to	Facility,					NA		
physiotherapy	14	moderately	individual	10	11 to 15	100%	100%		63%	N
OR control	13	severe					92%			

Kurtais 2008,		Mild to	Facility,						NR	NRM
treadmill	13	moderate	delivery	6	12 +	U (100%)^‡^	92%	Y		
walking OR			unclear +		home X					
			home,							
control	14		individual				86%			

Lehman 2005^a^,		Mild	Facility implied,					NA	NR	NRM
walk with	5		delivery unclear	2	5	100%	100%			
verbal cues OR control	6						100%			

Mak 2008,		Mild to	Facility						NR	NRM
cued sit to	21	moderate	Implied,	4	4	100%	90%	U		
stand OR			delivery							
OR exercise	21		unclear	4	6	100%	90%	U		
control	18						78%			

Marchese	U	Mild to	Facility,				U	NR	NR	NRM
2000,	20	moderate	individual +							
physiotherapy	total		home,	6	18 +	U (100%)‡				
OR			individual		home X					
physiotherapy				6	18 +	U (100%)‡				
with cues					home X					

Miyai 2000*,		Mild to	Facility,					NA	NR	NRM
treadmill	5	moderate	individual	4	6	100%	100%			
walking OR										
physiotherapy	5			4	6	100%	100%			

Miyai 2002,		Mild to	Facility,						NR	NRM
treadmill	12	moderate	individual	4	6	100%	92%	N		
walking OR										
physiotherapy	12			4	6	100%	75%	N		

Morris 2009,		Mild to	Facility,					NA		NRM
movement	14	moderate	individual	2	Up to 12	100%	100%		88% of	
strategies OR									maximum	
									sessions	
exercises	14			2	Up to 12	100%	100%		81% of	
									maximum	
									sessions	

Müller 1997,		Mild to	Location and					NR	NR	NRM
behavioural	15	moderate	delivery	10	30	100%	U			
therapy OR			unclear							
nonspecific	14			10	30	100%	U			
exercises and information										

Nieuwboer 2007* (also as [61, 73]),		Mild to moderate	Home, individual							Partial— no falls wearing activity monitor
cueing	76			3	4.5	100%	99%	N	100%	
training OR										
control	77						100%			

Pacchetti		Mild to	Facility, group				U	NR	NR	NRM
2000,		moderate								
physiotherapy	16			13	19.5	100%				
OR										
music therapy	16			13	26	100%				

Palmer 1986,		Mild to	Facility, group					NA	NR	NRM
exercise OR	7	moderately		12	36	100%	100%			
seated karate	7	severe		12	36	100%	100%			

Pelosin 2010,		Mild to	Facility,					NA	NR	NRM
physiotherapy	9	moderate	delivery	4	12	100%	100%			
with FOG			unclear							
strategies OR										
physiotherapy	9			4	12	100%	100%			

Protas 2005,		Mild to	Facility,					NA		NRM
treadmill	9	moderate	individual	8	24	100%	100%		100%	
walking										
and step										
training OR										
control	9						100%			

Qutubuddin		Mild to	Facility,							NRM
2007,		moderate	delivery							
computerized	12		unclear +	8	18	20%	75%	U	U	
dynamic			home,							
posturography			individual							
OR										
physiotherapy	10			8	18	20%	60%	U	U	

Ridgel 2009,		Mild to	Facility,					NA	NR	NRM
forced cycling	5	moderate	delivery	8	24	100%	100%			
OR			unclear							
self-paced	5			8	24	100%	100%			
cycling										

Sage 2009,		Mild to	Facility,							NRM
aerobic	17	moderate	group	10 to 12	18	100%	76%	Y	86.8%^†^	
exercise OR										
SAFEx OR	21			10 to 12	20 to 34	100%	86%	Y	92.9%^†^	
control	15						100%			

Sage 2010,		Mild to	Facility,					NA		NRM
SAFEx OR	15	moderate	group	12	36	100%	100%		100%	
non-SAFEx	14			12	36	100%	100%		100%	

Schenkman		Mild to	Location							
1998,		moderate	unclear,							
exercise OR	27		individual	10 to 13	22.5 to 30	100%	85%	U	100%†	N
control	24						96%			

Schmitz-		Mild to	Facility group						NR	NRM
Hübsch 2006,		moderately	+ home							
Qigong OR	32	severe	individual	24	16 + home X	66%	91%	Y		
control	24						87.5%			

Smania 2010,		Moderate	Facility,						NR	NRM
balance	33	to	individual	7	17.5	100%	85%	Y		
training OR		moderately								
general	31	severe		7	17.5	100%	87%	Y		
exercises										

Stallibrass		Mild to	Facility,							NRM
2002,		moderate	individual							
Alexander	32			12	16	100%	91%	Y	99%^†^	
technique										
OR										
massage OR	31			12	16		93%		97%^†^	
control	30						100%			

Tamir 2007,		Mild to	Facility,						NR	Partial—
exercise + imagery	12	moderate	delivery	12	24 +	U (100%)^‡^	92%	U		2 cardiac
OR			unclear +		home X					problems
exercise	11		home, individual	12	24 + home X	U (100%)^‡^	91%	U		

Thaut 1996,		Mild to	Home,					NA		
walk with	15	moderate	individual	3	10.5	14%	100%		100%	Y—1 fall
auditory cues										
OR										
walk without	11			3	10.5	14%	100%		100%	
cues OR										
control	11						100%			

Toole 2000,		Mild to	Facility,						NR	NRM
exercise OR	6	moderately	delivery	10	30	100%	67%	N		
control	5	severe	unclear				60%			

Toole 2005,	U	Mild to	Facility,				NR	NR	99%	NRM
treadmill	23	moderately	delivery	6	6	100%			overall	
walking with	total	severe	unclear							
body weight										
support OR										
treadmill				6	6	100%				
walking with										
weights OR										
treadmill				6	6	100%				
walking										

Yang 2010,		Mild to	Facility,							
downhill	16	moderate	delivery	4	6	100%	94%	Y	100%^†^	N
treadmill			unclear							
walking OR										
conventional	17			4	6	100%	88%	Y	100%^†^	N
therapy										

Yousefi 2009,		Mild to	Facility,					NA	NR	NRM
exercise OR	12	moderate	group	10	40	100%	100%			
education	12			10	40		100%			

Supervision: the number of intervention sessions supervised expressed as a percentage of the number of sessions prescribed; retention: the number of participants who completed the trial (i.e., undertook a post-intervention assessment but excluding follow-up) expressed as a percentage of the number of participants who began the trial; adherence: the number of intervention sessions participants attended expressed as a percentage of the number of intervention sessions prescribed; Y: yes; N: no; NA: not applicable; U: unclear—insufficient information to categorize; NR: not reported; NRM: not reported to be monitored; X: exercise; FOG: freezing of gait; SAFEx: sensory attention focused exercise; *cross-over trial;^ †^data only for participants who completed the trial;^ ‡^data only for the facility component of the intervention;^a^part 2 of trial only.

**Table 2:** Dropout reasons when related to the intervention.

First author and year	Dropout reason	Number of participants
Allen 2010	Did not want to do the intervention	1
Ashburn 2007	Falls (but not during intervention)	1
Blackinton 2002	Safety concerns	1
Braun 2011	Imagery too confronting	1
Burini 2006	Poor adherence to exercise;	2
	back pain	1
de Bruin 2010	No access to necessary equipment	1
Dereli 2010	Did not want to do the intervention	1
Hackney 2008 (also as [54])	Exercise not intense enough;	1
	transport problems	2
Hackney 2009 (also as [54])	Knee pain;	1
	transport problems	2
Hackney 2010	Travel distance;	2
	classes too fatiguing;	1
	lack of interest	1
Hirsch 2003	Inguinal hernia	1
Kurtais 2008	Poor adherence to exercise	1
Sage 2009	Time commitment	4
Schmitz-Hubsch 2006	Uncomfortable in the group;	1
	uncomfortable with Qigong	1
Smania 2010	Uncooperative	4
Stallibrass 2002	Could not travel	1
Yang 2010	Low motivation;	1
	transport problems	1
